# Physical Fitness and Military Efficiency

**Published:** 1914-09-12

**Authors:** 


### PHYSICAL FITNESS AND MILITARY EFFICIENCY.

### Are the Medical Tests for Recruits too Severe?

In normal and peaceful times few activities probably attract less popular attention than the recruiting of men for His Majesty's Army. It is one of the operations which the ordinary citizen leaves to the authorities; and, save for an occasional question in the House of Commons, no one seems to concern himself about it. So long as the military machine works quietly, the general public hardly asks how it is constructed and maintained.

In the past few weeks we have had to learn that we no longer live in normal and peaceful times, and the recruiting of the Army has sprung into the very forefront of popular attention. The need for men to be trained as soldiers is proclaimed in all our streets, and the recruiting sergeant is at our very doors. Inevitably these public operations awake the amateur adviser and critic, and we are far from denying that this person has his value. At the same time he must not be exalted above measure, and before we accept his conclusions these must be tested by knowledge and by expert judgment. One conclusion which has been proposed in certain quarters in reference to the present recruiting activity is that the medical tests applied to the candidates are unnecessarily severe. Men are rejected, it is said, because of some technical deficiency, the existence of which in no sense qualifies their value as fighting units. And the examining doctors are being blamed for their adherence to a standard which is described as the outcome of officialdom and red tape. Our old friend *Punch* has, in some measure, voiced this complaint by his picture of the candidate who, when rejected on account of the state of his teeth, protested that his ambition was not to eat the Germans but to shoot them.

We quite agree that in the circumstances some reasonable relaxation of the usual standard may well be allowed. Indeed, it would not surprise us to find that the military authorities were quite as much awake to this as are their critics, and we have no doubt at all that the examining doctors are acting in accordance with rules which have been carefully formulated by those who not only exercise authority, but also bear responsibility. We venture to suggest the necessity of bearing in mind this latter consideration. It is nothing less than a patriotic claim that in the stress of the present juncture a free measure of confidence shall be given to those who are charged with our national affairs. Further, it is obvious that the selection or rejection, as the case may be, of candidates for the Army cannot be left to the fancy or judgment of the individual examiner. There must be some rule. Let us be ready to allow that though a general rule, here as elsewhere, may produce individual disappointments and perhaps inconsistent results, yet a rule of some kind or other is in the circumstances absolutely necessary; and, further, let us be willing to believe that in all human probability the whole position has been adequately considered by those who at the moment stand at the head of our military affairs. This is not the time to discuss in detail the actual tests or standards which are being applied to the new recruits. But we do wish to say that the percentage of rejections, as reported in the public prints, ought not to be regarded as a proof that these tests and standards are too severe. These rejections are proportionately less than those recorded among men who enlist in times of peace, and we are satisfied that they are due, at least in the great majority of cases, to the necessities of the position.

In discussing this question it is necessary to remember that the natural disposition of the military authorities is to accept as many men as possible. But they want men who will be a source not of weakness, but of strength. And every man who, when sent to the front, breaks down from ill-health is an added burden, if not a danger, to the combatant forces. He disappoints expectation; he restricts freedom of movement; he helps to crowd the lines of communication; and he occupies a hospital bed which may well be wanted for a wounded comrade. It is physiological soundness and physical fitness alone which can endure the terrible hardships of the present campaign.

There is perhaps one aspect of the physical condition of the candidates now passing through the recruiting offices which may be urged on the authorities. It is whether rejection for the "front" should necessarily involve rejection for home service. Unless we are likely to suffer a serious invasion—a highly unlikely contingency—the conditions of the two services are by no means identical. Of course, obvious organic disease, possibly quite unsuspected by the man himself, closes the issue in both directions. But some slight deficiency in height or in chest measurement—the latter quite capable of improvement by training—surely need not, in the case of a candidate otherwise suitable, exclude a man from the chance of home defence. Even the standard of dentition need not be too rigid, for the food supplies of the soldiers who are guarding our coasts are much less abnormal than those of a mobile expeditionary force. In regard to the latter, every experienced person knows that the state of the teeth is of very high importance; the portion of its anatomy on which an army marches is proverbial. Hence we offer no suggestion in favour of mitigating the rigour on which the authorities insist in selecting men who are to be sent abroad. But we do venture to ask whether it is not possible to find amongst those rejected for this honour a certain proportion of men who may be able to serve the common cause at home. They are, be it remembered, willing volunteers. Unfit for the first line, some of them may possibly do effective and honourable duty in the second. Whether this individual point has been under discussion or not, we do not know. But we think it well worthy of consideration.

